# Short-Term Outcomes of Lower-Extremity Surgical Revascularization for Chronic Limb-Threatening Ischemia Among Yemeni Patients: A Prospective Observational Study

**DOI:** 10.7759/cureus.117253

**Published:** 2026-09-30

**Authors:** Nabeel Y Al-Modwahi, Ali M Fadhel, Al-Ezy M Hamoud, Yahya A Al-Modwahi, Emad A Halboob, Aref Al-Hashedi, Mohammed A Al-Mnari, Milad A Al-Motawakill

**Affiliations:** 1 Department of Vascular Surgery, Faculty of Medicine and Health Sciences, Sana’a University, Sana'a, YEM; 2 Department of Cardiac Surgery, Al-Thawra Modern General Hospital, Sana’a, YEM; 3 Department of Vascular Surgery, Al-Thawra Modern General Hospital, Sana'a, YEM

**Keywords:** ankle-brachial index, chronic limb-threatening ischemia, graft patency, limb salvage, lower extremity revascularization, open surgical revascularization, prospective observational study, vascular surgery, vascuqol-6, yemen

## Abstract

Background: Chronic limb-threatening ischemia (CLTI) carries a high risk of amputation and mortality. We evaluated short-term clinical, hemodynamic, and quality-of-life outcomes after open lower-extremity surgical revascularization in a Yemeni cohort.

Methods: This single-center prospective observational cohort study was conducted at Al-Thawra Modern General Hospital, Sana'a, Yemen. We enrolled 39 consecutive adults with CLTI (Rutherford categories 4-6) who were selected for and considered fit to undergo open lower-extremity surgical revascularization between December 2022 and September 2024; follow-up was completed in December 2024. The study protocol was reviewed and approved by the Al-Thawra Hospital Ethics Committee (approval no. 33-2026), and written informed consent was obtained from all participants. The study was not registered. This was an exploratory consecutive cohort; no formal sample-size calculation was performed, and no contemporaneous endovascular, nonoperative, or primary-amputation comparator group was included. Primary endpoints were graft patency and limb salvage at three months. Secondary outcomes were secondary patency, postoperative complications, and changes in ankle-brachial index (ABI) and Vascular Quality of Life Questionnaire-6 (VascuQoL-6) scores. Paired comparisons used Wilcoxon signed-rank tests; Fisher's exact tests were used for categorical comparisons.

Results: The mean age was 60.9 years (SD 9.7), and 30 patients (76.9%) were male. Diabetes mellitus and hypertension were present in 34 (87.2%) and 30 (76.9%) patients, respectively. Nineteen patients (48.7%) had ischemic rest pain, and 20 (51.3%) had tissue loss. Great saphenous vein (GSV) and polytetrafluoroethylene (PTFE) grafts were used in 28 (71.8%) and 11 (28.2%) patients, respectively. At three months, primary patency was 84.6% (33/39 grafts; 95% CI 70.3%-92.8%), secondary patency was 89.7% (35/39 grafts; 95% CI 76.4%-95.9%), and limb salvage was 89.7% (35/39 patients; 95% CI 76.4%-95.9%). Given the small cohort, the confidence intervals around these estimates were relatively wide. Among the 35 patients with limb preservation, mean ABI increased from 0.35 (SD 0.11) to 0.55 (SD 0.12; mean difference 0.21, 95% CI 0.19-0.23; p<0.001), and mean VascuQoL-6 score increased from 10.3 (SD 2.9) to 16.6 (SD 4.0; mean difference 6.3, 95% CI 5.6-7.1; p<0.001). Complications occurred in nine patients (23.1%). In an exploratory unadjusted subgroup analysis, GSV use was associated with higher primary patency than PTFE use (92.9% vs 63.6%, p=0.042).

Conclusion: In this single-center prospective cohort, open surgical revascularization was feasible and was associated with 89.7% limb salvage and 84.6% primary patency at three months. Improvements in ABI and VascuQoL-6 were observed among patients with limb preservation. Autologous GSV use was associated with higher primary patency; this finding requires confirmation in larger, adjusted studies.

## Introduction

Peripheral arterial disease (PAD) is a major global health challenge. In 2010, an estimated 202 million people were living with PAD worldwide, 69.7% of whom resided in low- and middle-income countries (LMICs) [1]. Chronic limb-threatening ischemia (CLTI) is the most severe clinical presentation of PAD and is defined by the presence of PAD in combination with ischemic rest pain, gangrene, or lower-limb ulceration persisting for more than two weeks [2]. CLTI is associated with substantial risks of limb loss and mortality and with impaired health-related quality of life [2].

PAD may be under-recognized in the Middle East despite the high prevalence of major cardiovascular risk factors, including diabetes, hypertension, and smoking [3]. In a population-based survey in Sana'a, the crude prevalence of known diabetes was 6.57% [4]. A systematic review and meta-analysis of Yemeni patients with type 2 diabetes estimated pooled prevalences of hypertension and hyperlipidemia at 36.9% and 56.6%, respectively [5]. At Al-Thawra Modern General Hospital, a retrospective audit of patients admitted with diabetic foot ulcers found an amputation rate of 54.7%; a history of PAD was among the independent risk factors for amputation [6]. In Yemen, the available published lower-limb vascular data are limited and consist largely of retrospective reports and diabetic foot amputation audits; prospective outcome data following surgical revascularization for CLTI are scarce.

Both endovascular and open surgical revascularization have complementary roles in the management of CLTI. The choice between open and endovascular revascularization should ideally be individualized according to patient risk, limb severity, anatomic complexity, conduit availability, and patient preference [2,7]. However, endovascular revascularization was not routinely available at our center during the study period; therefore, this cohort specifically evaluated outcomes after open lower-extremity surgical revascularization. In the BEST-CLI trial, among patients with an adequate single-segment great saphenous vein (GSV), initial surgical bypass reduced the composite outcome of major adverse limb events or death compared with endovascular revascularization [8].

Accordingly, this prospective observational study aimed to describe short-term clinical, hemodynamic, and quality-of-life outcomes after open lower-extremity surgical revascularization among Yemeni patients with CLTI selected for and considered fit for open surgery. The prespecified primary endpoints were three-month primary graft patency and limb salvage; secondary endpoints were secondary patency, postoperative complications, and changes in ankle-brachial index (ABI) and Vascular Quality of Life Questionnaire-6 (VascuQoL-6) scores. No contemporaneous comparator group was included.

## Materials and methods

Study design and setting

We conducted a single-center prospective observational cohort study at the Department of Vascular Surgery, Al-Thawra Modern General Hospital, Sana'a, Yemen. Consecutive eligible patients were enrolled between December 2022 and September 2024, and three-month follow-up was completed in December 2024. The study protocol was reviewed and approved by the Al-Thawra Hospital Ethics Committee (reference no. 33-2026; approval letter dated 1 December 2022). The Ethics Committee confirmed that the protocol was in accordance with applicable ethical principles and guidelines. Written informed consent was obtained from all participants before enrollment. The study was not registered. This was an exploratory study, and no formal sample-size calculation was performed. No contemporaneous endovascular, nonoperative, or primary-amputation comparator group was included.

Patient selection

Eligible patients were adults with symptoms and clinical signs of CLTI who were scheduled for primary open lower-extremity surgical revascularization. CLTI was defined in accordance with the Global Vascular Guidelines as objectively documented atherosclerotic peripheral artery disease in combination with ischemic rest pain, non-healing lower-extremity ulceration, or gangrene persisting for more than two weeks [2].

Primary open lower-extremity surgical revascularization was defined as the first open revascularization procedure performed on the index limb. Eligible procedures included supragenicular or infragenicular femoropopliteal bypass, femorodistal bypass, femoral interposition bypass, and combined femoral endarterectomy with patch angioplasty plus femorofemoral bypass. Inclusion criteria were age 18 years or older, Rutherford clinical category 4, 5, or 6 CLTI, and a clinical decision that the patient was suitable for open surgical revascularization for limb salvage. Patients were excluded if they presented with acute limb ischemia, had undergone previous ipsilateral surgical or endovascular revascularization, had severe uncorrectable systemic comorbidities that precluded general or regional anesthesia, or declined participation.

During the study period, 45 patients were screened for eligibility. Six were excluded: three because of acute limb ischemia, two because they were unfit for surgery, and one because consent was declined. Therefore, 39 consecutive patients were enrolled and underwent primary open lower-extremity surgical revascularization. No enrolled patient was lost to the three-month follow-up. At three months, 35 patients had limb preservation, and four had undergone major amputation; the 35 patients with limb preservation were included in the paired hemodynamic and quality-of-life analyses. Patient flow is shown in Figure 1.

**Figure 1:**
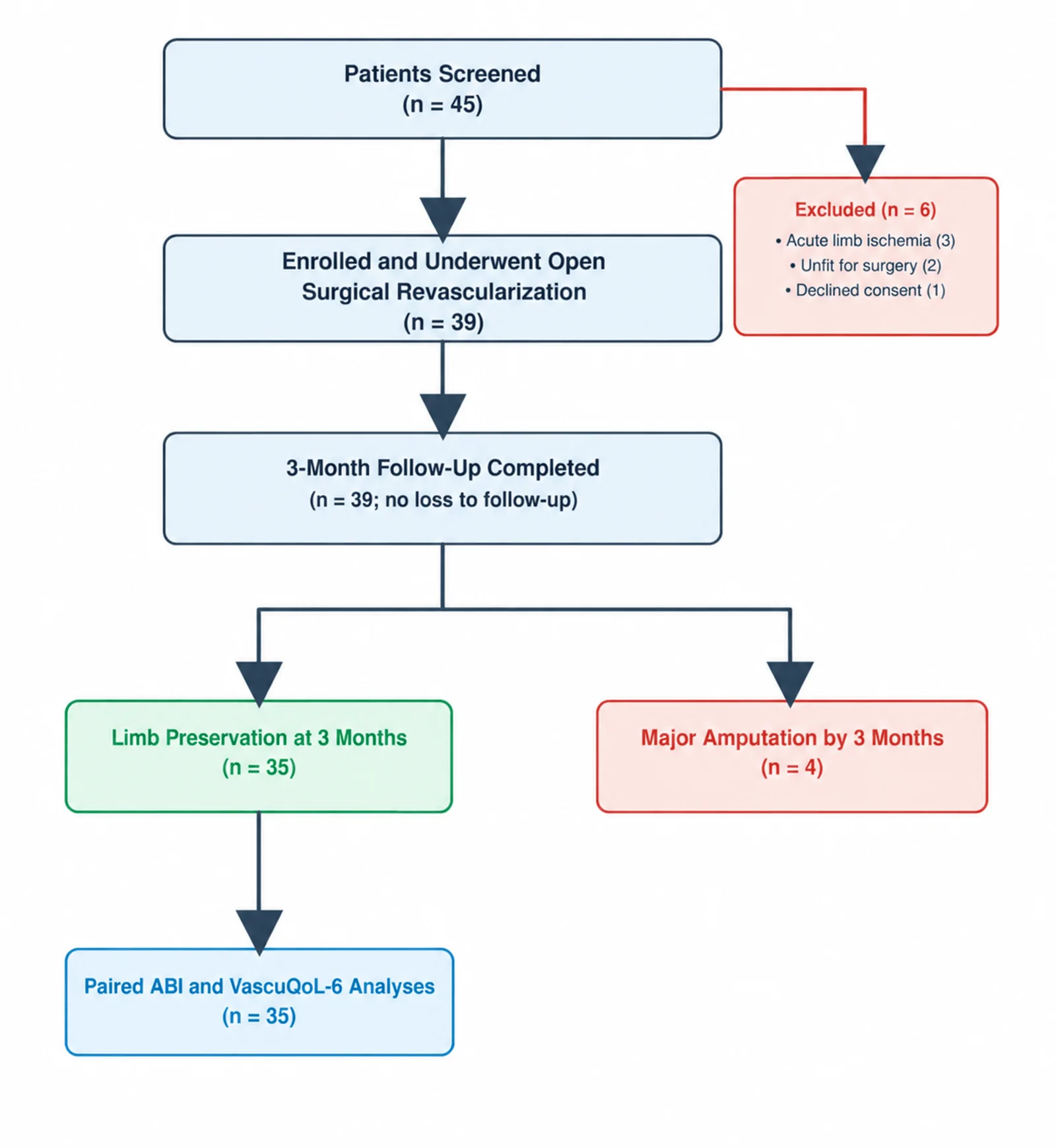
Study flow diagram showing screening, exclusions, enrollment, three-month follow-up, limb outcomes, and patients included in the paired ABI and VascuQoL-6 analyses ABI: Ankle-Brachial Index; VascuQoL-6: Vascular Quality of Life Questionnaire-6

Because enrollment was restricted to patients deemed fit for open surgery, this cohort may not represent all patients presenting with CLTI, including those unsuitable for surgery or managed nonoperatively.

Preoperative assessment

All enrolled patients underwent a comprehensive preoperative clinical and vascular evaluation. Available demographic data, medical history, and cardiovascular risk factors were recorded. Disease severity was clinically staged using the Rutherford classification system [9]. Anatomical mapping of arterial lesions was performed using preoperative duplex ultrasonography and/or computed tomography angiography (CTA). Femoropopliteal lesions were categorized according to the 2007 TransAtlantic Inter-Society Consensus II (TASC II) classification [10]. Infrapopliteal lesions were categorized according to the 2015 TASC below-the-knee anatomic classification [11]. For each limb, the target tibial artery was defined according to the planned revascularization pathway. The infrapopliteal TASC category was assigned from preoperative imaging according to lesion morphology and length, extent of occlusion, calcification, collateral visualization, and the severity of disease in the other tibial arteries.

Baseline hemodynamic status was assessed by measuring the ABI in all enrolled patients. After at least five minutes of supine rest, an appropriately sized pneumatic cuff selected according to limb circumference was used. Systolic pressures were obtained with a handheld continuous-wave Doppler probe at the dorsalis pedis and posterior tibial arteries of each limb and at both brachial arteries. ABI was calculated as the higher of the dorsalis pedis or posterior tibial systolic pressure divided by the higher brachial systolic pressure. At least two Doppler measurements were obtained at each site in all patients; additional readings were obtained when the initial reading or Doppler signal was uncertain. Measurements were performed by the study investigator, a vascular surgery resident.

Health-related quality of life was assessed using the six-item VascuQoL-6, a disease-specific instrument with total scores ranging from 6 to 24, where higher scores indicate better quality of life [12]. The questionnaire was administered orally in Arabic using a study-specific Arabic translation prepared for this study. No forward-backward translation, pilot testing, or formal assessment of reliability or validity was undertaken. Accordingly, this was not a validated Arabic version of VascuQoL-6, and the questionnaire findings should be interpreted as exploratory patient-reported outcomes. Toe pressures and toe-brachial indices were unavailable and were not assessed.

Although active smoking status was recorded, complete standardized data on baseline HbA1c, renal function, symptom duration, preoperative medication use, and cumulative smoking exposure were not available for all participants and were therefore not included in the analyses.

Surgical procedure

Open surgical revascularization was performed under general or regional (spinal or epidural) anesthesia by a dedicated vascular surgery team. The choice of arterial inflow and outflow was based on preoperative imaging and intraoperative clinical assessment. For femoral interposition bypass, an interposition graft was placed from the common femoral artery to the superficial femoral artery in the same limb. The combined procedure consisted of common femoral endarterectomy with patch angioplasty followed by femorofemoral crossover bypass.

When suitable, the GSV was the preferred conduit and was harvested as a reversed vein graft. A GSV was considered suitable when it had a diameter of at least 3 mm and was free of significant varicosities, sclerosis, or thrombosis. Polytetrafluoroethylene (PTFE) grafts were used when an adequate GSV was unavailable due to prior stripping, insufficient caliber, or poor conduit quality.

Systemic heparinization with 5,000 IU intravenously was administered before arterial clamping. Perioperative antibiotic therapy was individualized according to each patient's clinical condition; the specific agent, dose, and duration were not standardized and could continue postoperatively when clinically indicated. No routine intraoperative Doppler assessment, duplex ultrasonography, or completion angiography was performed. Graft adequacy was assessed clinically by the operating team. Postoperative duplex ultrasonography was obtained within hours after surgery in most patients, whereas postoperative CTA was performed selectively when clinically indicated.

Postoperative management

Postoperatively, all patients received dual antiplatelet therapy with aspirin 100 mg and clopidogrel 75 mg daily for the first month. Thereafter, long-term single antiplatelet therapy with either aspirin or clopidogrel was selected according to individual clinical considerations. Statin therapy was initiated or continued in all patients using either atorvastatin or rosuvastatin; the agent and dose were individualized.

Glycemic control was optimized in collaboration with the endocrinology service, with a target HbA1c level of less than 8%. Wound care consisted of clean dressings tailored to the clinical needs of the wound. Debridement was performed when clinically indicated, including in the presence of devitalized tissue, clinical infection, or a non-healing wound.

Duplex ultrasonography surveillance was completed for all 39 patients at the one-month and three-month follow-up visits; no patients were lost to follow-up, and no surveillance data were missing. Duplex examinations were performed and interpreted by a radiology specialist. Grafts were considered patent when continuous flow was demonstrated throughout the graft without occlusion. Stenosis was assessed using Doppler velocity measurements, color-flow findings, and spectral waveform characteristics, according to the radiology specialist’s routine clinical interpretation. Specific numerical velocity thresholds for stenosis were not retained as standardized analytical data; therefore, no uniform peak systolic velocity or velocity-ratio cutoff was applied in the present analysis.

Postoperative follow-up and endpoints

Patients were monitored during their inpatient stay and attended scheduled follow-up clinic visits at one month and three months postoperatively. The primary endpoints at three months were primary graft patency and limb salvage. Primary graft patency was defined as uninterrupted graft patency without any secondary intervention to maintain or restore patency. Secondary patency was defined as graft patency following an intervention to restore patency after graft occlusion. Limb salvage was defined as avoidance of major amputation of the revascularized limb. Major amputation was defined as any amputation above the level of the ankle.

Patency status was adjudicated by the study team, comprising vascular surgery consultants, specialists, and residents, using duplex ultrasonography reports, serial clinical assessment, and operative reports of thrombectomy or any other graft-related reintervention. Duplex surveillance was performed at one and three months. The same standardized handheld continuous-wave Doppler ABI measurement technique used at baseline was repeated at the three-month follow-up.

Secondary endpoints were secondary graft patency, change in ABI from baseline to three months, change in VascuQoL-6 score over the same period, and postoperative complications. Postoperative complications, including surgical-site infection, graft thrombosis, bleeding, and perioperative mortality, were recorded from the index operation through completion of the three-month follow-up. Perioperative mortality was defined as death occurring during the index hospitalization or within 30 days after surgery.

Paired ABI and VascuQoL-6 analyses were restricted to the 35 patients with limb preservation at three months. The four patients who underwent major amputation were excluded because postoperative ABI could not be measured in the revascularized limb; VascuQoL-6 analyses were restricted to the same subgroup for comparability. Consequently, these paired analyses represent a survivor subgroup and may overestimate hemodynamic and quality-of-life improvement when applied to the full cohort.

Statistical analysis

All statistical analyses were performed using Python version 3.12 (Python Software Foundation, Wilmington, DE, USA), using Pandas version 2.2 for data manipulation and SciPy version 1.18 for statistical testing. This was an exploratory consecutive cohort study; all eligible patients treated during the recruitment period were included, and no formal sample-size calculation was performed.

Baseline characteristics were summarized for the full enrolled cohort (n = 39). Continuous variables were expressed as means ± standard deviations (SD), and categorical variables as absolute frequencies and percentages. For paired preoperative and three-month postoperative measurements, mean within-patient differences were calculated from individual paired observations. Because the distributions of paired differences were non-normal on Shapiro-Wilk testing, 95% confidence intervals (CIs) for mean differences were estimated using nonparametric percentile bootstrap resampling, and statistical significance was assessed using the Wilcoxon signed-rank test. Mean values with SDs were reported for descriptive purposes. CIs for proportions, including primary patency, secondary patency, and limb-salvage rates, were calculated using the Wilson score method.

Exploratory unadjusted comparisons by conduit type were performed using Fisher’s exact test because of the small subgroup sample sizes. No adjustment for multiple comparisons was performed. All tests were two-sided, with p < 0.05 considered statistically significant.

Patients who underwent major amputation during follow-up (n = 4) were excluded from the paired ABI analysis because ABI could not be measured in the revascularized limb after amputation. VascuQoL-6 analyses were restricted to the same limb-preservation subgroup to maintain a common paired analysis population. Accordingly, paired ABI and VascuQoL-6 analyses were performed in 35 patients and describe outcomes among patients with limb preservation at three months rather than the entire enrolled cohort. No imputation-based sensitivity analysis was performed because post-amputation ABI was structurally unavailable and imputing paired outcomes would require unsupported assumptions. Time-to-event analyses, including Kaplan-Meier estimates, were not performed because exact dates of graft failure, reintervention, major amputation, and censoring were not available in the analytic dataset. Outcomes were therefore reported at the prespecified three-month endpoint.

## Results

Baseline demographic and clinical characteristics

The final cohort comprised 39 patients who underwent primary open lower-extremity surgical revascularization. Mean age was 60.9 years (SD 9.7), and 30 patients (76.9%) were male. Diabetes mellitus was present in 34 patients (87.2%), hypertension in 30 (76.9%), and active smoking in 15 (38.5%). Dilated cardiomyopathy, coronary artery disease, and heart failure were present in eight (20.5%), seven (17.9%), and four (10.3%) patients, respectively. Baseline ABI and VascuQoL-6 scores for the full enrolled cohort were 0.33 (SD 0.11) and 9.8 (SD 3.1), respectively.

Nineteen patients (48.7%) had Rutherford category 4 ischemic rest pain, 10 (25.6%) had category 5 minor tissue loss, and 10 (25.6%) had category 6 major tissue loss. Femoropopliteal lesions were classified as TASC II A in seven patients (17.9%), B in 10 (25.6%), C in 19 (48.7%), and D in three (7.7%). Infrapopliteal TASC classification was assigned only to the 20 patients with infrapopliteal disease on preoperative imaging: two (10.0%) had category A lesions, three (15.0%) category B, five (25.0%) category C, and 10 (50.0%) category D. The remaining 19 patients had no infrapopliteal disease and were therefore not classified. Baseline characteristics are summarized in Table 1.

**Table 1:** Baseline demographic, clinical, and anatomic characteristics of the study cohort (n = 39) * Percentages for the 2015 TASC below-the-knee classification are calculated among the 20 patients with infrapopliteal disease. Lesions were categorized according to the 2015 TASC below-the-knee anatomic classification. Baseline ABI and VascuQoL-6 values were calculated for the full enrolled cohort (n = 39), including the four patients who subsequently underwent major amputation. TASC: TransAtlantic Inter-Society Consensus; VascuQoL-6: Vascular Quality of Life Questionnaire-6

Characteristic	n or mean ± SD	Percentage (%)
Age (years)	60.9 ± 9.7	-
Male	30	76.9
Female	9	23.1
Cardiovascular risk factors		
Diabetes mellitus	34	87.2
Hypertension	30	76.9
Active smoking	15	38.5
Dilated cardiomyopathy (DCM)	8	20.5
Coronary artery disease (CAD)	7	17.9
Heart failure (HF)	4	10.3
Rutherford classification		
Category 4 (rest pain)	19	48.7
Category 5 (minor tissue loss)	10	25.6
Category 6 (major tissue loss)	10	25.6
TASC II femoropopliteal lesions		
Type A	7	17.9
Type B	10	25.6
Type C	19	48.7
Type D	3	7.7
2015 TASC below-the-knee classification (n = 20)*		
Type A	2	10.0
Type B	3	15.0
Type C	5	25.0
Type D	10	50.0
Baseline hemodynamic and quality-of-life measures		
Ankle-Brachial Index (ABI)	0.33 ± 0.11	-
VascuQoL-6 Score (range 6-24)	9.8 ± 3.1	-

Procedural details

Open revascularization procedures comprised femorodistal bypass in 14 patients (35.9%), infragenicular femoropopliteal bypass in 11 (28.2%), supragenicular femoropopliteal bypass in five (12.8%), femoral interposition bypass in seven (17.9%), and combined common femoral endarterectomy with patch angioplasty plus femorofemoral crossover bypass in two (5.1%). A reversed autologous GSV was used in 28 grafts (71.8%) and PTFE in 11 grafts (28.2%). Available procedural characteristics and intraoperative assessment are summarized in Table 2.

**Table 2:** Available procedural characteristics and intraoperative assessment (N = 39) Values are n (%) unless otherwise stated. *Femoral interposition bypass refers to an ipsilateral common femoral artery-to-superficial femoral artery graft. ^†^ This procedure comprised common femoral endarterectomy, patch angioplasty, and femorofemoral crossover bypass.

Characteristic	Value
Open surgical procedure	
Femorodistal bypass	14 (35.9%)
Infragenicular femoropopliteal bypass	11 (28.2%)
Supragenicular femoropopliteal bypass	5 (12.8%)
Femoral interposition bypass*	7 (17.9%)
Common femoral endarterectomy with patch angioplasty plus femorofemoral crossover bypass^†^	2 (5.1%)
Conduit	
Reversed great saphenous vein	28 (71.8%)
Polytetrafluoroethylene graft	11 (28.2%)
Vein selection criteria	Great saphenous vein diameter ≥3 mm, without significant varicosities, sclerosis, or intraluminal thrombosis
Systemic heparinization	5,000 IU intravenously before arterial clamping
Anesthesia	General or regional anesthesia (spinal/epidural); anesthesia-specific counts were not available
Antibiotic regimen	Administered preoperatively; antibiotic selection and postoperative continuation were individualized according to the patient’s clinical condition
Routine intraoperative completion imaging	Not performed; no routine intraoperative Doppler, duplex ultrasonography, or completion angiography was used
Intraoperative technical assessment	Clinical intraoperative assessment by the operating vascular team
Postoperative duplex surveillance	Performed in all patients at one and three months
Target tibial vessel, runoff score, operative time, and estimated blood loss	Not available in a standardized, analyzable form

Clinical and hemodynamic outcomes

At three months, primary graft patency was 84.6% (33/39 grafts; 95% CI 70.3%-92.8%). Six grafts thrombosed; two were treated successfully with surgical thrombectomy, resulting in secondary patency of 89.7% (35/39 grafts; 95% CI 76.4%-95.9%). Limb salvage was 89.7% (35/39 patients; 95% CI 76.4%-95.9%), and four patients (10.3%) underwent major amputation. Given the small cohort, the CIs around these primary outcome estimates were relatively wide.

Among the 35 patients with limb preservation at three months, mean ABI increased from 0.35 (SD 0.11) to 0.55 (SD 0.12); the mean paired difference was 0.21 (95% CI 0.19-0.23; Wilcoxon signed-rank p < 0.001) (Figure 2A). Mean VascuQoL-6 score increased from 10.3 (SD 2.9) to 16.6 (SD 4.0); the mean paired difference was 6.3 (95% CI 5.6-7.1; Wilcoxon signed-rank p < 0.001) (Figure 2B). These paired hemodynamic and quality-of-life outcomes are summarized in Table 3.

**Figure 2:**
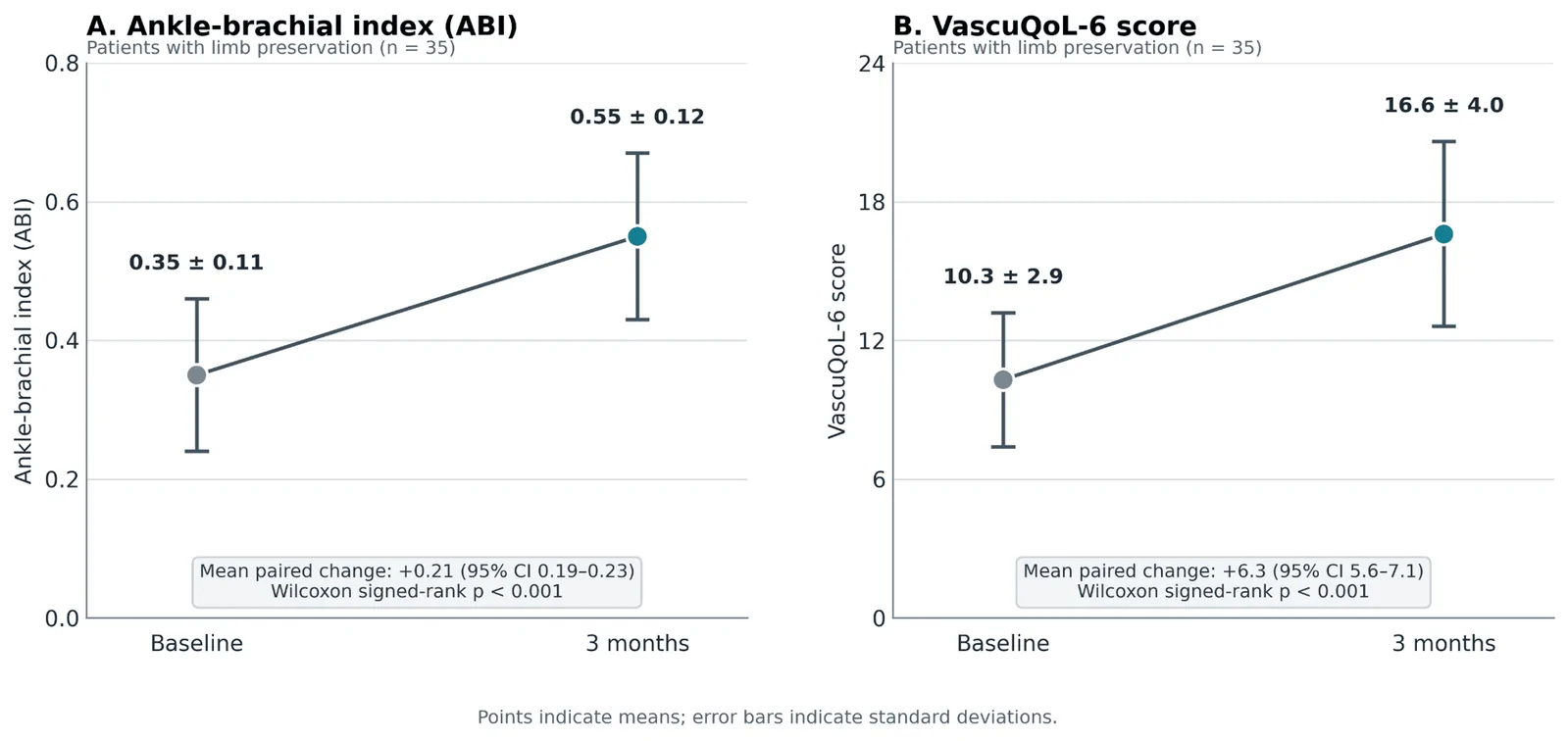
Changes in ABI and VascuQoL-6 scores from baseline to three months among patients with limb preservation (n = 35) Points indicate means, and error bars indicate SDs. Paired analyses included 35 patients with limb preservation at three months; four patients who underwent major amputation were excluded. The mean paired change in ABI was 0.21 (95% CI 0.19-0.23; Wilcoxon signed-rank P < 0.001), and the mean paired change in VascuQoL-6 score was 6.3 (95% CI 5.6-7.1; P < 0.001). ABI: ankle-brachial index; CI: confidence interval; n: number of patients; SD: standard deviation; VascuQoL-6: Vascular Quality of Life Questionnaire-6

**Table 3:** Hemodynamic and quality-of-life outcomes preoperatively and at three months postoperatively * Values are mean ± SD. Paired analyses included 35 patients with limb preservation at three months; four patients who underwent major amputation were excluded. Ninety-five percent CIs for mean differences were estimated using nonparametric percentile bootstrap resampling. P-values were derived using the Wilcoxon signed-rank test. ABI: ankle-brachial index; CI: confidence interval; SD: standard deviation; VascuQoL-6: Vascular Quality of Life Questionnaire-6

Outcome parameter	Preoperative value	Postoperative (three months)	Mean difference (95% CI)	p-value*
ABI	0.35 ± 0.11	0.55 ± 0.12	0.21 (0.19 to 0.23)	<0.001
VascuQoL-6 Score (range 6-24)	10.3 ± 2.9	16.6 ± 4.0	6.3 (5.6 to 7.1)	<0.001

Postoperative complications and exploratory subgroup analysis

Postoperative complications occurred in nine patients (23.1%). Combined acute graft thrombosis and deep surgical-site infection occurred in four patients (10.3%), all of whom underwent major amputation. Isolated graft thrombosis occurred in two patients (5.1%) and was treated with surgical thrombectomy. Other recorded complications were isolated wound infection, postoperative hematoma, and wound bleeding requiring re-exploration, each in one patient (2.6%). No perioperative deaths occurred, and no deaths were recorded during the three-month follow-up.

In an exploratory, unadjusted analysis, primary graft patency was 92.9% (26/28) with GSV and 63.6% (7/11) with PTFE (Fisher’s exact p = 0.042). Limb salvage was 96.4% (27/28) with GSV and 72.7% (8/11) with PTFE (p = 0.060). Table 4 summarizes these exploratory comparisons.

**Table 4:** Exploratory unadjusted comparison of three-month outcomes by conduit type * Fisher's exact test. Percentages are calculated within each conduit group; analyses are exploratory and unadjusted, and no adjustment for multiple comparisons was performed. CI: confidence interval; GSV: great saphenous vein; PTFE: polytetrafluoroethylene

Clinical outcome	Autologous GSV (n = 28)	Synthetic PTFE (n = 11)	p-value*
Primary graft patency	26 (92.9%; 95% CI: 77.4-98.0)	7 (63.6%; 95% CI: 35.4-84.8)	0.042
Limb salvage rate	27 (96.4%; 95% CI: 82.3-99.4)	8 (72.7%; 95% CI: 43.4-90.3)	0.060

A descriptive comparison of baseline characteristics by three-month limb outcome is presented in Table 5. Given the small number of major amputations (n = 4), no hypothesis testing was performed for this comparison.

**Table 5:** Descriptive baseline characteristics by three-month limb outcome Values are mean ± SD or n (%). The comparison is descriptive only; no hypothesis testing was performed because of the small number of major amputations. ABI: ankle-brachial index; VascuQoL-6: Vascular Quality of Life Questionnaire-6

Characteristic	Limb preservation (n = 35)	Major amputation (n = 4)
Age, years	61.1 ± 10.0	59.8 ± 7.3
Male sex	27 (77.1%)	3 (75.0%)
Diabetes mellitus	31 (88.6%)	3 (75.0%)
Hypertension	27 (77.1%)	3 (75.0%)
Active smoking	13 (37.1%)	2 (50.0%)
Rutherford category 4	19 (54.3%)	0 (0.0%)
Rutherford category 5	8 (22.9%)	2 (50.0%)
Rutherford category 6	8 (22.9%)	2 (50.0%)
Baseline ABI	0.35 ± 0.11	0.23 ± 0.05
Baseline VascuQoL-6 score	10.3 ± 2.9	6.0 ± 0.0

## Discussion

Our prospective observational cohort provides early estimates of outcomes after open lower-extremity surgical revascularization in 39 consecutive adults with CLTI selected for and considered fit to undergo open surgery at a single referral center in Yemen. At three months, primary graft patency was 84.6% (33/39; 95% CI, 70.3%-92.8%), secondary patency was 89.7% (35/39; 95% CI, 76.4%-95.9%), and limb salvage was 89.7% (35/39; 95% CI, 76.4%-95.9%). Six grafts thrombosed, and successful surgical thrombectomy in two patients accounted for the difference between primary and secondary patency. No deaths occurred during follow-up. Among the 35 patients with limb preservation, ABI and VascuQoL-6 scores increased. However, because this study had no comparator group, these results do not establish the causal effect or comparative effectiveness of open surgery.

The case mix indicates a substantial burden of disease: 87.2% of patients had diabetes mellitus, 51.3% had tissue loss, and 10 of the 20 patients with infrapopliteal disease had TASC category D lesions. These findings should not be interpreted as evidence that poor glycemic control or delayed presentation caused greater disease severity, because standardized baseline and longitudinal HbA1c data and the interval from symptom onset to presentation were not available for full-cohort analysis. The relatively wide CIs around the principal outcome estimates also reflect the limited precision of this small cohort.

Guidelines recommend individualized selection of surgical or endovascular revascularization according to patient risk, limb severity, anatomic complexity, conduit availability, and patient preference [2,13]. Randomized trials do not support a universal strategy: in cohort 1 of BEST-CLI, surgery reduced the composite incidence of major adverse limb events or death compared with endovascular therapy among patients with an adequate single-segment GSV, whereas no statistically significant difference was observed in cohort 2, in which an alternative conduit was required [8]. In contrast, BASIL-2 reported better amputation-free survival with a best-endovascular-treatment-first strategy among patients requiring infrapopliteal revascularization, largely because fewer deaths occurred in the endovascular group [14]. These studies enrolled different populations, compared treatment strategies, and reported longer-term composite outcomes; therefore, they are context rather than direct benchmarks for the present three-month patency and limb-salvage estimates.

The GSV versus PTFE comparison was exploratory and unadjusted. Primary patency was higher with GSV than with PTFE (92.9% vs. 63.6%; p = 0.042), whereas the difference in limb salvage was not statistically significant (96.4% vs. 72.7%; p = 0.060). This direction is consistent with recommendations favoring suitable autogenous vein for popliteal or infrapopliteal bypass when available [2,13] and with prior evidence suggesting better patency with GSV than prosthetic grafts in selected settings [15,16]. However, PTFE was used when an adequate GSV was unavailable; procedures and distal targets were heterogeneous, and the groups were not adjusted for runoff, anastomotic level, disease severity, or other confounders. With only 11 PTFE grafts and no adjustment for multiple exploratory comparisons, this finding should be considered hypothesis-generating only.

Among patients with limb preservation, mean ABI increased from 0.35 ± 0.11 to 0.55 ± 0.12, with a mean paired difference of 0.21 (95% CI, 0.19-0.23; p < 0.001). This supports improved ankle-level perfusion, although the postoperative mean ABI remained abnormal. The analysis excluded the four patients who underwent major amputation and therefore does not represent the hemodynamic experience of the full cohort. Toe pressure, toe-brachial index, and other local perfusion measures were unavailable. This is important in a predominantly diabetic cohort because medial arterial calcification can falsely elevate ankle pressures, and toe-based measurements are preferred when available [2].

VascuQoL-6 is a validated, disease-specific instrument for peripheral artery disease [12]. Among the 35 patients with limb preservation, the mean score increased from 10.3 ± 2.9 to 16.6 ± 4.0, with a mean difference of 6.3 points (95% CI, 5.6-7.1; p < 0.001). Although this numerical change exceeded the 4.5-point individual-level threshold proposed for substantial clinical benefit one year after revascularization in patients with critical limb ischemia [17], that threshold cannot be applied directly to this study. Assessment occurred at three months, the Arabic VascuQoL-6 version was a study-specific translation that was not formally validated, and patients who underwent major amputation were excluded from the paired analysis. The observed change therefore reflects improvement within the limb-preservation subgroup rather than clinically meaningful benefit for every participant or for the full cohort [18].

Nine patients (23.1%) experienced a reported postoperative complication. Four had both acute graft thrombosis and deep surgical-site infection and underwent major amputation; two additional isolated graft thromboses were successfully treated with thrombectomy. Although diabetes has been associated with a higher short-term risk of wound infection after lower-extremity revascularization [19], only five participants in this cohort did not have diabetes, and no predictor analysis was performed. The study cannot therefore determine the independent contribution of diabetes, infection, thrombosis, or other factors to graft loss or amputation. Similarly, the absence of deaths in 39 patients during three months of follow-up does not establish negligible operative or cardiovascular risk.

This study prospectively documents outcomes from an open surgical program in a setting where endovascular revascularization was not routinely available. Its strengths include consecutive enrollment, complete three-month follow-up, scheduled duplex surveillance, CIs for the principal proportions, and assessment of graft patency, limb preservation, hemodynamics, and disease-specific quality of life. The findings provide a preliminary local benchmark for similarly constrained settings, but do not demonstrate superiority over another treatment strategy.

Several limitations warrant emphasis. This was a small, single-center referral cohort with no formal sample-size calculation; participants were selected for and considered fit to undergo open surgery, which limits precision and generalizability to the broader CLTI population. The absence of a contemporaneous endovascular, nonoperative, or primary-amputation comparator prevents estimation of relative treatment effects. Follow-up was limited to three months, and exact event dates were unavailable for time-to-event analyses; consequently, long-term graft durability, repeated intervention, wound healing, functional outcomes, cardiovascular events, and survival cannot be adequately assessed. Procedures and target levels were heterogeneous, and the conduit comparisons were unadjusted and susceptible to confounding by indication, while detailed procedural variables, including runoff score, target tibial vessel, anesthesia-specific counts, operative time, and estimated blood loss, were not available in a standardized, analyzable form. Complete standardized data on HbA1c, renal function, symptom duration, preoperative medication use, and smoking pack-years were also unavailable for the full cohort, precluding more detailed risk adjustment. Conditioning ABI and VascuQoL-6 analyses on limb preservation excluded patients with the poorest limb outcome and may overestimate cohort-level improvement. Finally, the absence of Wound, Ischemia, and foot Infection (WIfI) staging, Global Limb Anatomic Staging System (GLASS) classification, toe-based perfusion measures, and a formally validated Arabic VascuQoL-6 version limits comparison with contemporary CLTI studies and risk-adjusted interpretation [2].

Accordingly, these findings are best interpreted as preliminary estimates of short-term graft patency, limb preservation, and within-subgroup improvement after open revascularization in a selected Yemeni cohort, rather than evidence of long-term durability or superiority over another strategy. Future multicenter studies should incorporate contemporary limb and anatomic staging, validated Arabic patient-reported measures, toe-based perfusion testing, standardized wound-healing and functional endpoints, adjusted conduit analyses, time-to-event methods, and longer follow-up.

## Conclusions

Among 39 consecutive Yemeni adults with CLTI selected for and considered fit to undergo open lower-extremity surgical revascularization, three-month primary graft patency was 84.6% (33/39; 95% CI, 70.3%-92.8%) and limb salvage was 89.7% (35/39; 95% CI, 76.4%-95.9%). ABI and VascuQoL-6 scores improved among the 35 patients with limb preservation; these paired analyses do not represent the full cohort. The observed higher primary patency with GSV than with PTFE arose from a small, unadjusted exploratory comparison and is hypothesis-generating only, not evidence of a causal conduit effect. These preliminary, single-center, short-term findings provide a local benchmark for selected patients fit for open surgery, but are limited by the small sample, heterogeneous procedures, absence of a comparator group, and survivor-selection bias in the paired outcomes. Larger multicenter studies should incorporate WIfI and GLASS staging, validated Arabic patient-reported measures, toe-based perfusion assessment, and longer follow-up.
